# Phylogeography of a successful aerial disperser: the golden orb spider *Nephila *on Indian Ocean islands

**DOI:** 10.1186/1471-2148-11-119

**Published:** 2011-05-09

**Authors:** Matjaž Kuntner, Ingi Agnarsson

**Affiliations:** 1 Institute of Biology, Scientific Research Centre, Slovenian Academy of Sciences and Arts, Novi trg 2, P. O. Box 306, SI-1001 Ljubljana, Slovenia; 2 Department of Entomology, National Museum of Natural History, Smithsonian Institution, NHB-105, PO Box 37012, Washington, D.C. 20013-7012, USA; 3 Department of Biology, University of Puerto Rico - Rio Piedras (UPR-RP), San Juan, PR, 00931, Puerto Rico

## Abstract

**Background:**

The origin and diversification patterns of lineages across the Indian Ocean islands are varied due to the interplay of the complex geographic and geologic island histories, the varying dispersal abilities of biotas, and the proximity to major continental landmasses. Our aim was to reconstruct phylogeographic history of the giant orbweaving spider (*Nephila*) on western Indian Ocean islands (Madagascar, Mayotte, Réunion, Mauritius, Rodrigues), to test its origin and route of dispersal, and to examine the consequences of good dispersal abilities for colonization and diversification, in comparison with related spiders (*Nephilengys*) inhabiting the same islands, and with other organisms known for over water dispersal. We used mitochondrial (COI) and nuclear (ITS2) markers to examine phylogenetic and population genetic patterns in *Nephila *populations and species. We employed Bayesian and parsimony methods to reconstruct phylogenies and haplotype networks, respectively, and calculated genetic distances, fixation indices, and estimated clade ages under a relaxed clock model.

**Results:**

Our results suggest an African origin of Madagascar *Nephila inaurata *populations via Cenozoic dispersal, and the colonization of the Mascarene islands from Madagascar. We find evidence of gene flow across Madagascar and Comoros. The Mascarene islands share a common 'ancestral' COI haplotype closely related to those found on Madagascar, but itself absent, or as yet unsampled, from Madagascar. Each island has one or more unique haplotypes related to the ancestral Mascarene haplotype. The Indian Ocean *N. inaurata *are genetically distinct from the African populations.

**Conclusions:**

*Nephila *spiders colonized Madagascar from Africa about 2.5 (0.6-5.3) Ma. Our results are consistent with subsequent, recent and rapid, colonization of all three Mascarene islands. On each island, however, we detected unique haplotypes, consistent with a limited gene flow among the islands subsequent to colonization, a scenario that might be referred to as speciation in progress. However, due to relatively small sample sizes, we cannot rule out that we simply failed to collect Mascarene haplotypes on Madagascar, a scenario that might imply human mediated dispersal. Nonetheless, the former interpretation better fits the available data and results in a pattern similar to the related *Nephilengys*. *Nephilengys*, however, shows higher genetic divergences with diversification on more remote islands. That the better disperser of the two lineages, *Nephila*, has colonized more islands but failed to diversify, demonstrates how dispersal ability can shape both the patterns of colonization and formation of species across archipelagos.

## Background

Oceanic islands are convenient models for studying dispersal of biotas and for understanding how dispersal ability relates to speciation [1]. Questions concerning the origination and diversification of lineages across the Indian Ocean islands are fascinating because of the interplay of the complex geographic and geologic history of the islands, the varying dispersal abilities of local biotas, and the proximity to major continental landmasses, Africa and Asia, with dramatically different biotas. Madagascar, for example contains both the lineages of vicariant origin and those arriving via more recent Cenozoic dispersal, and a mixture of African, Asian, and even Australasian elements [2-4]. Some other smaller volcanic islands, in turn, only contain lineages that have dispersed there, but the source landmasses may be diverse including the above mentioned continents, as well as Madagascar, and other islands. The distribution and diversity of lineages across volcanic islands will, to a large degree, be a function of dispersal ability of lineages, with better dispersers occupying more of the islands. However, the fact that good dispersers occur on more islands does not necessarily mean that they are also more diverse across archipelagos. One of the key questions surrounding dispersal is to 'determine the impact of dispersal distances and [for spiders] ballooning propensity on gene flow and speciation..." [5]. Gene flow is more rapidly disrupted in poor dispersers because even narrow barriers can be effective isolators [6,7]. Hence, processes of allopatric speciation are expected to start operating earlier in poor dispersers.

**Figure 1:**
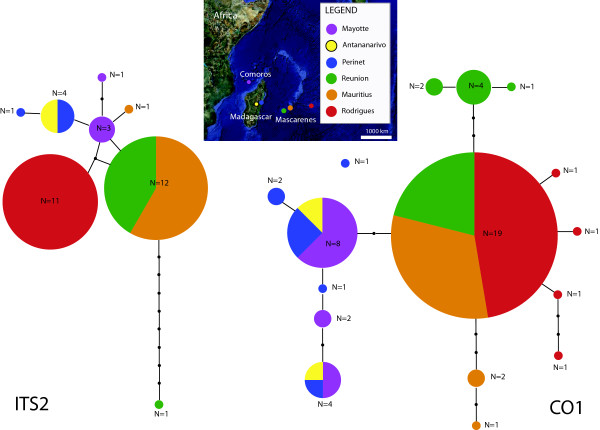
**Area of study with sampling localities and haplotype networks (ITS2, CO1) of *Nephila *populations**. The latter are consistent with one species inhabiting the islands of the western Indian Ocean, but in the process of speciating, e.g. on Rodrigues. Islands are colour coded, and the size of each haplotype is proportional to the number of individuals sharing it. Lines connect haplotypes through inferred substitution events (dots).

Many spiders are excellent dispersers, such that they have colonized and diversified across archipelagos worldwide. Therefore, spiders have played a prominent role as study organisms in the island diversification and biogeography [8-16]; however, none of these studies had a focus on the Indian Ocean apart from Madagascar and Comoros [10,17]. Here we study a lineage of an excellent disperser, the giant golden orb weaving spiders (genus *Nephila*), and compare and contrast its colonization route, as well as the distribution and diversity of this lineage with that of a poorer disperser, its sister lineage *Nephilengys *[18]. The giant golden orb weaving spiders (genus *Nephila*) are distributed pantropically and represent conspicuous elements of tropical terrestrial invertebrate faunas [19]. Compared with *Nephilengys, Nephila *species are much more widespread (Table 1) having colonized most land masses except the most remote oceanic islands such as Hawaii, Galapagos, and Polynesia. Precise mechanisms of *Nephila *dispersal are not documented; however, orb weavers generally disperse by wind travel termed 'ballooning' [5], which is likely also true for *Nephila *[20]. *Nephila *populations are found on most islands of the Indian Ocean, e.g. Madagascar, the Comoro chain, Aldabra and Seychelles, as well as the Mascarene archipelago, from Réunion through Mauritius to Rodrigues (Figure 1) [21].

**Table 1:** Major landmasses and islands occupied by *Nephil a *and *Nephilengys*

Landmasses and islands	*Nephila*	*Nephilengys*
North America	y	
Central America	y	
South America	y	y
Cuba	y	
Hispaniola	y	
Puerto Rico	y	
Lesser Antilles	y	
Eurasia	y	y
Africa	y	y
Cape Verde	y	
Sao Tome	y	y
Socotra	y	
Seychelles	y	y
Aldabra	y	y
Comoros (incl. Mayotte)	y	y
Madagascar	y	y
Réunion	y	y
Mauritius	y	y
Rodrigues	y	
Sri Lanka	y	y
Hainan	y	
Taiwan	y	
Japan	y	
Philippines	y	y
Singapore	y	y
Sumatra	y	y
Java	y	y
Sulawesi	y	y
Lesser Sundas	y	y
Moluccas	y	
New Guinea	y	y
New Britain	y	
Solomon Islands	y	
New Caledonia	y	
Australia	y	y
Tasmania	y	
New Zealand	y	
Vanuatu	y	
Fiji	y	
Tonga	y	
**TOTAL**	**40**	**19**

*Nephila *is much more widespread, which suggests it is a better disperser compared with its sister genus *Nephilengys*. Data from [18,21,61] and http://www.nephilidae.com.

**Figure 2:**
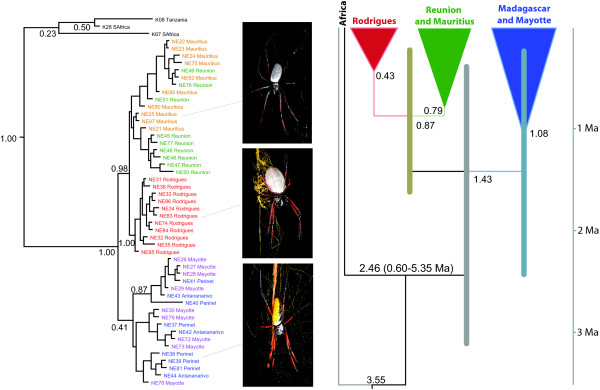
**Phylogeny of the Indian Ocean *Nephila *species supports the hypothesis of an African origin; this combined with the haplotype networks (Figure 1) is consistent with a dispersal east into the more remote islands**. Depicted are typical female morphologies from these islands. On the right are the results from the BEAST dating analysis, displaying estimated node ages of selected clades, bars represent 95% confidence intervals.

Recently, we investigated biogeographic and diversification patterns of *Nephilengys *in this region, and found that its diversity had been underestimated [18]. We found that the patterns of *Nephilengys *phylogenetic and population genetic structure indicate a Cenozoic colonization of Madagascar from Africa, with a subsequent colonization of, and diversification in, more remote islands. Thus, two of the three volcanic Mascarene islands, Réunion and Mauritius, harbour an endemic species each, whereas the populations on Mayotte (part of the Comoro chain) are intermixed with those on Madagascar. Compared to *Nephilengys, Nephila *inhabits one additional island in the Mascarenes, namely Rodrigues, a small and isolated volcanic island (109 km^2^, 560 km east of Mauritius; Figure 1). On Rodrigues, *Nephila *is thought to be represented by an endemic species, *N. ardentipes *Butler, 1876, differing from *N. inaurata *in female habitus (Figure 2) and in other morphological features [19]. Other target populations purportedly belong to *N. inaurata *(Walckenaer, 1842), a species widespread from the eastern African coast to the islands of the Indian Ocean [19,21,22]. However, the species status of both *N. inaurata *and *N. ardentipes *requires testing with molecular data.

Within this study, we were interested in a comparison of biogeography and diversification patterns between *Nephila *and *Nephilengys *on the same islands in the Indian Ocean. Because they represent sister genera [19,22] they are of comparable ages, and thus likely to follow comparable geological histories. We predicted that *Nephila *would follow a colonization pattern from Africa on to Madagascar and further onto remoter islands, as does *Nephilengys *[18]. Secondly, we aimed to compare patterns of diversification in both genera by an analysis of genetic structure of populations on each island, with a particular focus on the status of *N. ardentipes *on Rodrigues. Third, we aimed to compare the modes and success of dispersal between *Nephila *and *Nephilengys *on these islands. Given that *Nephila *is globally much wider spread than *Nephilengys *(Table 1), we predicted that *Nephila *would inhabit more islands in the Indian Ocean, and would exhibit a higher level of gene flow among these islands. Finally, we were interested in comparing the biogeographic patterns of *Nephila *on the Indian Ocean islands with those of other aerially dispersed organisms, in order to detect common causes [23].

## Results

TCS reconstructed haplotype networks from both genes suggest either gene flow persisted until recently among populations on all islands (Figure 1), or a recent simultaneous colonization of all three Mascarene islands took place from Madagascar. None of these networks, however, connect to the *N. inaurata *populations on the African mainland in the 95% connection limit of TCS. Some haplotypes are shared between several islands, e.g. Réunion and Mauritius (ITS2), Mayotte and Madagascar (CO1), and Rodrigues, Mauritius and Réunion (CO1). The ITS2 haplotype found on Rodrigues is unique (Figure 1) and apparently represents a single six base pair deletion event and is treated as such in the ITS2 network.

Bayesian and parsimony phylogenetic analyses (Figure 2) of both genes independently, as well as a combined analysis, suggest that the Indian Ocean *Nephila *are monophyletic and sister to *N. inaurata *from mainland Africa. Further, we find a clade with the populations from Réunion, Mauritius, and Rodrigues, separate from a mixed Madagascar plus Mayotte clade (with very low support), or grade. The population from Rodrigues is recovered monophyletic, and that clade receives strong support (Figure 2).

F_ST _estimates among each of these islands show strong geographical genetic structuring, yet are consistent with a recent gene flow within and between the islands or a recent colonization of the Mascarenes (F_ST _= 0.502, p < 0.001). Maximal uncorrected genetic distance (CO1) between the populations in Madagascar and the Mascarenes was about 1.5% and between Réunion and Mauritius was 0.6%, while the maximal uncorrected genetic distance between Madagascar and the African *N. inaurata *was about 5.5%. These *Nephila *genetic distances are significantly lower than those in the genus *Nephilengys *(Table 2).

**Table 2:** Differences in maximal uncorrected genetic distances (COI) between the populations of an excellent disperser (*Nephila*, this paper) and an intermediate disperser, *Nephilengys *[18] on the islands of the western Indian Ocean

	***Nephila***	***Nephilengys***
Africa -- Madagascar	5.5%	7-9.5%
Madagascar -- Mascarenes	1.5%	5.3%
Réunion -- Mauritius	0.6%	3-4%

BEAST analysis estimates the age of the node separating African and island populations at 2.46 (0.60 to 5.35) Ma, and the ages of other, less inclusive nodes, at less than 2 Ma (Figure 2). These estimates of node ages clearly rule out Gondwanan origin of *Nephila *on Madagascar and the neighbouring islands.

Together, the phylogenies, haplotype networks, fixation indices, and genetic divergences with clade dating are consistent with an African origin of the Madagascar populations via Cenozoic dispersal, and with a subsequent and rapid dispersal from Madagascar to the Mascarenes. However, the shallow genetic divergences indicate either occasional periods of gene flow, or a very recent colonization of the Mascarenes, such that populations on all islands are best characterized as belonging to a single taxonomic species, *N. inaurata*. Due to limited sampling we cannot rule out that haplotypes found in Mascarenes also occur on Madagascar, thus further sampling is necessary to test the observed patterns.

## Discussion

We studied phylogeographic patterns of the giant orb web spider, *Nephila*, on the islands of the Indian Ocean, and investigated how these compare with related spiders and with other organisms known for long range, over ocean dispersal. We sampled *Nephila *on the islands Mayotte, Madagascar, Réunion, Mauritius and Rodrigues, and tested the route and timing of their oceanic origin, genetic structure among the islands, and the status of the enigmatic Rodrigues population, which is sometimes referred to as a separate species, *N. ardentipes *[19,21]. We found phylogenetic and population genetic structures based on nuclear (ITS2) and mitochondrial markers (CO1) to be consistent with a dispersal model from the African mainland, and a recent and rapid colonization of the Mascarenes, or a recent termination of gene flow between Madagascar and Mascarene populations. Taxonomically, all targeted populations are thus best circumscribed as one species, *N. inaurata*, which invalidates *N. ardentipes *as a species. However, the Rodrigues population is monophyletic and has a unique ITS2 haplotype, and the other Mascarene islands have also started accumulating unique haplotypes. This might be consistent with a 'speciation in progress' scenario. Another possibility cannot be completely ruled out, that all haplotypes also occur on Madagascar but that we failed to sample Mascarene haplotypes from Madagascar. However, we find this unlikely given the structure of the data, the number of unique haplotypes on the Mascarenes, and the fact that *Nephila *is not synanthrophic in the area and thus not likely human transported.

*Nephila *has colonized at least 40 major islands and land masses globally (Table 1), and many species within this clade are extremely widespread. The best examples are the American *N. clavipes *spanning from North America through Central and South America into Argentina, the African mainland *N. fenestrata *and *N. senegalensis *covering most of the continent, and the Australasian *N. pilipes*, which ranges from India to Solomon Islands, and from Japan to Australia [20,21,24]. Such large ranges are unusual for invertebrates, and hint at excellent dispersal abilities and ecological success. Although not empirically observed to balloon [5,25], we find ballooning, most likely at the earliest ontogenetic stages, to be the most logical explanation for *Nephila *colonizing remote islands. Since *Nephila *spiders are not synanthropic (as are, e.g., some species of the sister genus, *Nephilengys*), the travel among islands by human assistance is unlikely. This conclusion is supported by the timing of inferred colonization of the Indian Ocean islands, which vastly predates human settlement at slightly over 2000 years ago.

Perhaps the apparently high dispersal abilities have limited *Nephila *diversification by maintaining gene flow among even geographically distant populations. The taxonomy of *Nephila *is well studied and the genus is hypothesized to contain only 14 species globally [[21,22]; this paper]. Although its sister genus, *Nephilengys*, is even less diverse globally, it invites a direct comparison within the region of study. We also studied the speciation patterns of the *Nephilengys *populations on the same islands [18], and found molecular, biogeographical and morphological evidence for three species: *Ng. livida *inhabiting Madagascar and Comoros, *Ng. borbonica *endemic to Réunion, and *Ng. dodo *from Mauritius. *Nephila *and *Nephilengys *show comparable biogeographic histories - both lineages occupied Madagascar from Africa between 2 and 5 Ma (although the upper bound estimates for *Nephilengys *are 13 Ma), and other smaller islands more recently, after which *Nephilengys *diversified through a lack of gene flow, while *Nephila *diverged less, with the only deep divergencies occurring between the African mainland and the population across the Indian Ocean as a whole (Table 2). Combined, the comparable timing of initial colonization of the Indian Ocean by both lineages and the subsequent speciation and lack of gene flow in *Nephilengys *rule out the potential human assisted travel to remote islands in *Nephilengys*.

The third nephilid spider genus inhabiting some of these islands is *Clitaetra*, known from Madagascar, Comoros, Sri Lanka and mainland Africa [26,27]. *Clitaetra *are much smaller spiders that inhabit forest trees, and probably are poor dispersers. Consequently, Comoros are inhabited by an endemic species, as apparently the belt of sea between Mayotte and Madagascar 300 km wide presents enough of a barrier to prevent gene flow. Judging by the number of landmasses occupied by each lineage, *Clitaetra *is a poor disperser and *Nephila *a very successful one, while *Nephilengys*, as an intermediate disperser, is the most diverse of the three across the Indian Ocean archipelago. Better dispersers can colonize more islands, but also require larger distances to effectively prevent gene flow, while poor dispersers only rarely reach less isolated islands with few opportunities to diversify [6,14]. Does this simple model hold for other organisms on these islands?

Recent literature provides compelling evidence for the origin of the majority of the Indian Ocean island biotas via Cenozoic dispersal rather than via vicariant origin on ancient Gondwanan landmasses [[2,4,18]; but, see [3]]. Thus, the best explanation for the origin of most biotas on the islands is that their ancestors must have arrived relatively recently, when the landmasses were in, or close to today's position, having mainly arrived from Africa, but also with elements from Asia and Australasia. The modes of dispersal must be either aerial, rafting on ocean, or a mix of both, and in some cases, assisted by human transport [28]. We argue that *Nephila *and *Nephilengys *spiders fall in the category of aerial dispersers, with no evidence of human transport among islands in the region.

Aerial transport, either active or passive with wind, is probably the best understood mode of dispersal. Several groups of flying animals have colonized the Indian Ocean islands and speciated there, but their origin varies. Logically, the oceans present the least of a barrier to birds and bats. For example, oscine passerines dispersed from Australia to Asia, and on over the Indian Ocean to Africa, where they radiated [29]. Parrots reached the Mascarenes from India [30], and *Triaenops *bats colonized Madagascar from Africa several times resulting in several independent lineages there [31]. Another group of organisms that disperse by wind are flying insects; in allodapine bees for example, there is a pervasive pattern of African Miocene origin with several dispersal events onto Madagascar, to Asia, and to Australia [32]. These authors concluded that the bees possess the ability to cross large expanses of ocean via west drift wind, and did not exclude the potential of over water rafting over the Mozambique Channel between Africa and Madagascar. Apparently, for flying insects, the Mozambique Channel (just over 400 km wide) presents only a moderate barrier to dispersal. To dragonflies over ocean wind dispersal presents little difficulty as evidenced by wind assisted colonization of the Indian Ocean islands from Asia [33].

Terrestrial and freshwater groups, both of presumed lower dispersal abilities compared with aerial dispersers, have also occupied most of the islands that we studied, e.g. lizards [34-38] and frogs [28]. These groups probably used rafting on ocean as means of dispersal. Chameleons, once believed to be of Gondwanan origin on Madagascar, have in fact colonized Madagascar over the ocean where they subsequently radiated [34]. They then spread to Comoros and Seychelles, where they also speciated. One species is thought to have recently colonized Réunion where it has accumulated morphological differences from the source population in Madagascar. In each case, over water dispersal events occur frequently enough to allow colonization of several islands not followed by extinction, but rare enough such that colonization events immediately restrict gene flow and eventually lead to speciation. Coastal lizards (genus *Cryptoblepharus*) are globally distributed in Asia and the islands around Madagascar, where they diversified, then separately colonized the East African coast, the Comoros islands and Mauritius, but not Réunion or Rodrigues [38]. This suggests the occurrence of occasional over water dispersal, which is rare enough to lead to speciation even on islands separated by small bodies of water. *Phelsuma *geckos show higher speciation rates [35,36] with a species rich radiation confined to Madagascar, from where a colonization event to the Mascarenes is dated at 4-5 Ma, followed by speciation on all the islands: three endemic species are known from Réunion, five on Mauritius and three on Rodrigues, of which two are extinct [36]. These studies suggest that over ocean dispersal in lizards is possible but rare, and these relatively poor dispersing abilities facilitate speciation in the absence of recent gene flow.

The above examples of groups with good (aerial) dispersers versus moderate (rafting) dispersers provide us with the following comparison of radiation success: Birds as the best dispersers have colonized all the Mascarene islands, but have not radiated [4,30]. *Triaenops *fruit bats, also good dispersers, have repeatedly colonized Madagascar and adjacent islands in the relatively recent past, but remain species poor [31], presumably due to continuous gene flow. Among the best insect dispersers, dragonflies, colonized all the islands but only diversified very moderately [33]. Rafting dispersers, presumably of medium dispersal abilities, are present in almost all the islands, and exhibit some exceptionally diverse radiations (chameleons on Madagascar and *Phelsuma *geckos throughout the archipelago, see above). Terrestrial mammals and amphibians, presumably poor dispersers, are entirely absent from the Mascarenes [30], but have radiated in Madagascar after reaching it during rare dispersal events: lemurs, rodents, tenrecs and carnivores radiated on Madagascar (reviewed by [4]). The common pattern seems to be analogous to the *Nephila-Nephilengys-Clitaetra *example that we studied. Therefore, the model of intermediate dispersal abilities underlying diversification across archipelagos seems to be also supported by the data from organisms other than spiders. The well-known trend of good dispersers losing their dispersal ability subsequent to colonizing islands also may lead to speciation events, another example where somewhat reduced dispersal ability positively correlates with diversification across archipelagos. Of course, a broader comparison would be needed to better test the validity of this speciation model, which is beyond our scope here.

## Conclusions

Both the island area and the amount of gene flow between islands affect speciation rates on remote islands [7]. We summarize evidence for several groups of terrestrial organisms that have successfully dispersed over the Indian Ocean islands being borne with wind or carried on oceanic rafts. Yet the Mozambique Channel represents a significant barrier to gene flow for most of these colonizers resulting in subsequent speciation on Madagascar, and through the Indian Ocean islands. In orbweb spiders, ballooning is the best understood mode of dispersal, and although the literature fails to demonstrate *Nephila *dispersal via ballooning, we agree with other authors [20,25] that *Nephila *must do so. *Nephila *appears to disperse more readily to isolated islands than *Nephilengys*. We find evidence that either its populations in the remote Indian Ocean islands have maintained some limited gene flow with the remaining island populations until recently, or that they have colonized all three Mascarene islands very recently, such that *N. ardentipes *is best treated as synonymous with *N. inaurata*. On the other hand, in *Nephilengys*, dispersal to remote Mascarene islands resulted in speciation [18]. This mirrors the patterns of *Nephila *biogeography elsewhere in the tropics, e.g. in Australasia [20,39]. The global picture where *Nephila *is spread over more than twice the land masses compared to *Nephilengys *(Table 1) yet exhibits lower levels of genetic divergences among these islands compared to *Nephilengys *(Table 2), reinforces this conclusion. In sum, the patterns in two sister spider lineages, *Nephila *and *Nephilengys*, and in other taxa suggest that excellent dispersers may colonize more islands but diversify less. This hints at how dispersal ability can shape both the patterns of colonization and formation of species across archipelagos.

## Methods

*Nephila *specimens were collected in the field into 95% ethanol on the islands Mayotte, Réunion, Mauritius, Rodrigues and from two localities in Madagascar (Additional file 1). To test the origin of Indian Ocean *Nephila*, samples of *N. inaurata *were obtained from S and E Africa, in addition to outgroup *Nephila *species represented by the African *N. constricta *and *N. turneri *and the Australasian *N. pilipes *as the primary outgroup (Additional file 1). Voucher specimens will be deposited at the National Museum of Natural History, Smithsonian Institution.

We isolated DNA from each individual's leg with the QIAGEN DNAeasy Tissue Kit (Qiagen, Inc., Valencia, CA), and amplified fragments of one mitochondrial (COI) and one nuclear (ITS2) locus using the LCOI1490 [40] and C1-N-2776 [41] primer pair for COI, and the ITS-5.8S (FITS) and ITS-28S (RITS) pair [42] for ITS2. These genes were chosen as readily amplifiable markers that have been shown to be useful at shallow taxonomic levels [43-45], and that represent both nuclear and mitochondrial genomes. We used standard protocols [43-46] with 47°C annealing temperature for 30 cycles. The PCR products were sequenced by the Sequencing and Genotyping facility of the University of Puerto Rico. Sequences were submitted to Genbank (Additional file 1). Our inability to obtain both CO1 and ITS2 sequences for all terminals was likely due to amplification of *Nephila *symbionts by universal ITS2 primers.

Sequences were inferred using Phred and Phrap to read and assemble the reads, respectively [47,48] through the Chromaseq package test version 0.984 [49] in the evolutionary analysis program Mesquite 2.74 [50]. We ran Phred using default options, and used Phrap with options -qual_show 20 -vector_bound 0. We trimmed sequence ends in Chromaseq using a moving window analysis: the Wrst window of 10 bases within which at least 6 were above quality score 20 was used as the start or end of the sequence. If a site had secondary peaks at least 0.3 the height of the primary peak, it was treated as ambiguous. Subsequently the sequences were proofread by comparing them with the chromatograms by eye. For alignments we used ClustalW [51] via Mesquite, with gap opening and extension costs set at 24/6. For both COI and ITS2 the alignments were unambiguous, the former with no gaps, and the latter with only a few, unambiguously placed gaps. Further exploration of alignment parameters was therefore not necessary. The alignment of both genes is available as Additional file 2.

We constructed haplotype networks in a statistical parsimony framework using TCS [52]. We calculated population genetic structure in Arlequin 3.5 [53], and uncorrected genetic distances in Mesquite.

The appropriate substitution model was selected with jModeltest 0.1.1 [54] using the AIC criterion [55] to select among the 24 models implemented in MrBayes. The best models were HKY+Γ+I for COI and HKY+Γ for ITS2 [56]. Bayesian analysis of the two loci combined was performed using MrBayes V3.1.2 [57]. The characters of each partition were 1-1226 (COI) and 1227-1541 (ITS2). The Markov chain Monte Carlo was run with four chains for 10,000,000 generations, sampling the Markov chain every 1000 generations, and the sample points of the first 5,000,000 generations were discarded as ''burnin".

We estimated node ages using BEAST 1.6.1. under uncorrelated exponential relaxed clock model [58,59]. Prior to the analysis we pruned taxa with significant missing data. As a calibration point we set the geological age of Réunion to 2.1 Ma [60] as a normally distributed prior with mean of 1Ma and extremes of standard deviation reaching 2.1, thus in effect setting the maximal age of the most recent common ancestor of Réunion haplotypes.

## Authors' contributions

Both authors participated equally in all phases of this research, wrote the manuscript and approved it.

## Supplementary Material

Additional file 1 Specimen data for terminals used in the phylogenetic analysis, with GenBank accession numbers, except where sequence not available (-) or does not reach 200 base pair length (*). See also Additional file 2. Click here for file

Additional file 2 **Aligned concatenated data matrix**. Click here for file
