# Supplementary material for: Phylogeography of a successful aerial disperser: the golden orb spider Nephila on Indian Ocean islands
Source: BMC Evol Biol. 2011 May 9;11:119. doi: 10.1186/1471-2148-11-119 (PMC3098804; doi:10.1186/1471-2148-11-119)
Supplement: Additional file 1 — Specimen data for terminals used in the phylogenetic analysis, with GenBank accession numbers, except where sequence not available (-) or does not reach 200 base pair length (*). See also Additional file 2. [file 1471-2148-11-119-S1.DOC]

**Additional files**

**Additional file 1 -** Specimen data for terminals used in the phylogenetic analysis, with GenBank accession numbers, except where sequence not available (-) or does not reach 200 base pair length (*). See also Additional file 2.

| **Taxon code** | ***Nephila* species** | **Locality** | **Accession nu. COI** | **Accession nu. ITS2** |
| --- | --- | --- | --- | --- |
| K15_Nephila_pilipes | *pilipes* | Australia, Trinity Park | JF835935 | - |
| K112_Nephila_turneri | *turneri* | Ghana, Axim | JF835936 | JF835914 |
| K40_Nephila_constricta | *constricta* | DR Congo, Mayombe | JF835937 | - |
| K08_Tanzania | *inaurata* | Tanzania (no locality) | JF835938 | - |
| K67_SAfrica | *inaurata* | South Africa, Ndumo | JF835939 | - |
| K28_SAfrica | *inaurata* | South Africa, Pongola | JF835940 | - |
| NE21_Mauritius | *inaurata* | Mauritius | - | JF835924 |
| NE22_Mauritius | *inaurata* | Mauritius | JF835941 | - |
| NE23_Mauritius | *inaurata* | Mauritius | JF835942 | * |
| NE24_Mauritius | *inaurata* | Mauritius | JF835943 | - |
| NE25_Mauritius | *inaurata* | Mauritius | JF835944 | JF835927 |
| NE26_Mayotte | *inaurata* | Mayotte | JF835945 | * |
| NE27_Mayotte | *inaurata* | Mayotte | JF835946 | - |
| NE28_Mayotte | *inaurata* | Mayotte | JF835947 | JF835926 |
| NE29_Mayotte | *inaurata* | Mayotte | JF835948 | - |
| NE30_Mayotte | *inaurata* | Mayotte | JF835949 | - |
| NE31_Rodrigues | *inaurata/ardentipes* | Rodrigues | JF835950 | JF835921 |
| NE32_Rodrigues | *inaurata/ardentipes* | Rodrigues | JF835951 | JF835922 |
| NE33_Rodrigues | *inaurata/ardentipes* | Rodrigues | JF835952 | JF835923 |
| NE34_Rodrigues | *inaurata/ardentipes* | Rodrigues | JF835953 | JF835915 |
| NE35_Rodrigues | *inaurata/ardentipes* | Rodrigues | JF835954 | JF835916 |
| NE36_Rodrigues | *inaurata/ardentipes* | Rodrigues | JF835955 | JF835917 |
| NE37_Perinet | *inaurata* | Madagascar, Perinet | JF835956 | * |
| NE38_Perinet | *inaurata* | Madagascar, Perinet | JF835957 | JF835930 |
| NE39_Perinet | *inaurata* | Madagascar, Perinet | JF835958 | JF835931 |
| NE40_Perinet | *inaurata* | Madagascar, Perinet | JF835959 | - |
| NE41_Perinet | *inaurata* | Madagascar, Perinet | JF835960 | - |
| NE42_Antananarivo | *inaurata* | Madagascar, Antananarivo | JF835961 | * |
| NE43_Antananarivo | *inaurata* | Madagascar, Antananarivo | JF835962 | - |
| NE44_Antananarivo | *inaurata* | Madagascar, Antananarivo | - | JF835925 |
| NE45_Réunion | *inaurata* | Réunion | JF835963 | JF835932 |
| NE46_Réunion | *inaurata* | Réunion | JF835964 | - |
| NE47_Réunion | *inaurata* | Réunion | JF835965 | - |
| NE48_Réunion | *inaurata* | Réunion | JF835966 | * |
| NE49_Réunion | *inaurata* | Réunion | JF835967 | JF835933 |
| NE50_Réunion | *inaurata* | Réunion | JF835968 | JF835929 |
| NE51_Réunion | *inaurata* | Réunion | JF835969 | - |
| NE72_Mayotte | *inaurata* | Mayotte | JF835970 | * |
| NE73_Mayotte | *inaurata* | Mayotte | JF835971 | * |
| NE74_Rodrigues | *inaurata/ardentipes* | Rodrigues | JF835972 | JF835918 |
| NE75_Mauritius | *inaurata* | Mauritius | JF835973 | * |
| NE76_Réunion | *inaurata* | Réunion | JF835974 | * |
| NE77_Réunion | *inaurata* | Réunion | JF835975 | JF835934 |
| NE78_Mayotte | *inaurata* | Mayotte | JF835976 | - |
| NE79_Mayotte | *inaurata* | Mayotte | JF835977 | - |
| NE80_Mauritius | *inaurata* | Mauritius | JF835978 | - |
| NE81_Perinet | *inaurata* | Madagascar, Perinet | JF835979 | - |
| NE82_Mauritius | *inaurata* | Mauritius | JF835980 | * |
| NE83_Rodrigues | *inaurata/ardentipes* | Rodrigues | JF835981 | JF835919 |
| NE84_Rodrigues | *inaurata/ardentipes* | Rodrigues | JF835982 | * |
| NE85_Rodrigues | *inaurata/ardentipes* | Rodrigues | JF835983 | * |
| NE86_Rodrigues | *inaurata/ardentipes* | Rodrigues | JF835984 | JF835920 |
| NE87_Mauritius | *inaurata* | Mauritius | JF835985 | JF835928 |
| NE88_Mauritius | *inaurata* | Mauritius | JF835986 | * |

**Additional file 2 –** Aligned concatenated data matrix.

Submitted as Nexus file.
